# Spontaneous In‐Plane Anomalous Hall Response Observed in a Ferromagnetic Oxide

**DOI:** 10.1002/adma.202502624

**Published:** 2025-09-16

**Authors:** Shinichi Nishihaya, Yuta Matsuki, Haruto Kaminakamura, Hiroki Sugeno, Ming‐Chun Jiang, Yoshiya Murakami, Ryotaro Arita, Hiroaki Ishizuka, Masaki Uchida

**Affiliations:** ^1^ Department of Physics Institute of Science Tokyo Tokyo 152‐8551 Japan; ^2^ RIKEN Center for Emergent Matter Science 2‐1 Hirosawa Wako 351‐0198 Japan; ^3^ Department of Physics and Center for Theoretical Physics National Taiwan University Taipei 10617 Taiwan; ^4^ Department of Physics University of Tokyo 7‐3‐1 Hongo, Bunkyo‐ku Tokyo 113‐0033 Japan; ^5^ Toyota Physical and Chemical Research Institute Nagakute 480‐1192 Japan

**Keywords:** anomalous hall effect, in‐plane field effect, magnetic weyl semimetal, molecular beam epitaxy, orbital magnetization

## Abstract

Recent observation of anomalous Hall effect (AHE) induced by magnetic field or spin magnetization lying in the Hall deflection plane has sparked interest in diverse mechanisms for inducing the Hall vector component perpendicular to the applied magnetic field. Such off‐diagonal coupling, which is strictly constrained by symmetry of the system, provides new degrees of freedom for engineering Hall responses. However, spontaneous response as extensively studied for out‐of‐plane AHE remains unexplored. Here, in‐plane AHE in a typical ferromagnetic oxide SrRuO_3_ is elucidated. The (111)‐orientated ultrathin films with in‐plane easy axes of spin magnetization exhibit spontaneous AHE at zero field, which is intrinsically coupled to the in‐plane spin magnetization and controllable via its direction. Systematic measurements by varying azimuthal and polar field angles further reveal complex Hall responses shaped by higher‐order terms allowed by trigonal distortion of the films. These findings highlight versatile and controllable in‐plane Hall responses with out‐of‐plane orbital ferromagnetism.

## Graphical abstract

Observation of in‐plane anomalous Hall effect (AHE) is demonstrated in a typical ferromagnet SrRuO_3_. The (111)‐orientated ultrathin films with in‐plane easy axes exhibit spontaneous AHE at zero field, which is controllable via its intrinsic coupling to the in‐plane spin magnetization through higher order effects allowed by symmetry, revealing the versatile and controllable in‐plane Hall responses with out‐of‐plane orbital ferromagnetism.

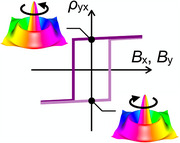

## 1 Introduction

**Figure 1:**
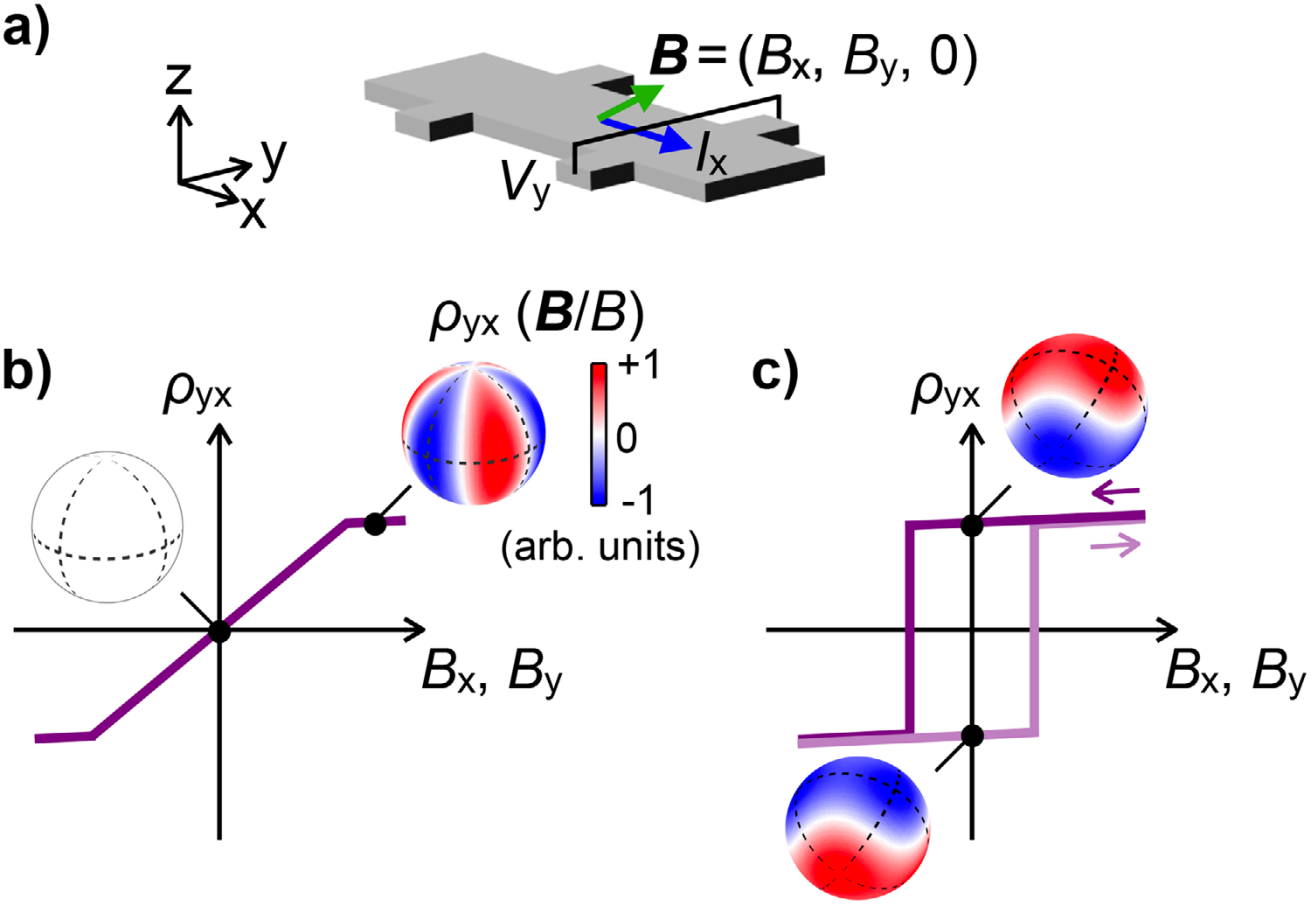
Anomalous Hall effect by in‐plane magnetic field. a) Measurement configuration of anomalous Hall effect (AHE) under the in‐plane magnetic field. A voltage *V*_y_ transverse to an electric current *I*_x_ can be generated depending not only on the value but also on the history of the in‐plane magnetic fields *B*_x_ and *B*_y_. b) For antiferromagnets, the Hall resistivity ρ_yx_ becomes zero at zero magnetic field, even in the case that large ρ_yx_ with three‐fold rotational symmetry around the z direction is induced by the in‐plane field. A case of (001)‐oriented trigonal systems is shown as an example of the field direction dependence of ρ_yx_. c) In hard ferromagnets, it is expected that ρ_yx_ spontaneously emerges even after turning off the in‐plane magnetic field. A case of (111)‐oriented cubic systems is exemplified.

Manipulation of spin and orbital magnetization by magnetic field is fundamental to diverse magnetotransport phenomena in magnets. The primary effect of the magnetic field is the Zeeman‐type coupling, where magnetic moments couple diagonally to the applied field.^[^ ^1^ ^]^ Recently, on the other hand, observation of Hall responses under the field applied within the Hall deflection plane has been reported (**Figure** **1**a).^[^ ^2^, ^3^, ^4^, ^5^, ^6^, ^7^ ^]^ In contrast to the conventional Hall effect induced by the out‐of‐plane magnetic field,^[^ ^8^, ^9^ ^]^ in‐plane field induced anomalous Hall effect (in‐plane AHE) can be interpreted as generation of the Hall vector component perpendicular to the in‐plane magnetic field. Namely, it is an off‐diagonal response to the magnetic field.

Following the theoretical proposals of in‐plane AHE and its quantization in topological materials,^[^ ^10^, ^11^, ^12^, ^13^, ^14^, ^15^, ^16^, ^17^ ^]^ experimental observations of in‐plane AHE in various materials have invoked interest in detailed mechanisms for realizing such off‐diagonal responses.^[^ ^2^, ^3^, ^4^, ^5^, ^6^, ^7^ ^]^ Among them, the in‐plane AHE observed for Weyl semimetals Fe_3_Sn_2_^[^ ^5^ ^]^ and EuCd_2_Sb_2_,^[^ ^6^ ^]^ which exhibits three‐fold symmetry for the in‐plane field rotation, has been understood as a consequence of band structure and quantum geometry modulations induced by the in‐plane magnetic field and the spin‐orbit coupling. The in‐plane field induced modulations are of purely intrinsic origins and thus strictly follows the crystal symmetry,^[^ ^16^, ^18^ ^]^ exhibiting the unique field direction dependence (Figure 1b). In particular, the nonzero in‐plane anomalous Hall conductivity with the in‐plane spin magnetization can be directly associated with contribution from the orbital magnetization component in the out‐of‐plane direction.^[^ ^19^, ^20^, ^21^ ^]^

So far research on in‐plane AHE has been limited to non‐magnetic,^[^ ^2^, ^3^, ^4^ ^]^ antiferromagnetic^[^ ^6^ ^]^ or soft‐magnetic materials^[^ ^5^, ^7^ ^]^ with negligible magnetic hysteresis. As shown in Figure 1c, hard ferromagnets potentially host anomalous Hall conduction and out‐of‐plane orbital ferromagnetism at zero field. Such a spontaneous response involves the off‐diagonal coupling between orbital and spin degrees of freedom, where the out‐of‐plane orbital magnetization is expected to be controlled via the in‐plane spin magnetization direction. Combination of the spontaneous response in ferromagnets and the unique off‐diagonal feature of in‐plane AHE provides new opportunities for engineering the Hall transport. On the other hand, spin canting or reorientation derived by magnetocrystalline or shape magnetic anisotropy in ferromagnets may complicate observations by inducing the out‐of‐plane spin magnetization component and conventional out‐of‐plane AHE. From this viewpoint, a ferromagnet with in‐plane easy axes is highly desired for elucidating the pure response coupled to the in‐plane spin magnetization.

Here, we study spontaneous in‐plane AHE observed in films of ferromagnetic perovskite oxide SrRuO_3_. Owing to the intrinsic mechanism, choosing materials such as SrRuO_3_ with the band structure hosting Berry curvature hotspots is expected to be beneficial for realizing large in‐plane AHE.^[^ ^22^, ^23^, ^24^ ^]^ (111)‐oriented ultrathin SrRuO_3_ films are epitaxially grown on the (111) SrTiO_3_ substrate with threefold rotational symmetry about the out‐of‐plane direction (see Section S1 and Figure S1, Supporting Information, for structural characterization). Throughout this work, pseudocubic expression is adopted for describing the crystal orientation of SrRuO_3_. A giant in‐plane AHE comparable to the out‐of‐plane AHE is observed in the SrRuO_3_ ultrathin films, where the anomalous Hall conduction persists at zero field and can be controlled via the in‐plane spin magnetization direction. Detailed measurements by varying azimuthal and polar angles of the field confirm that the SrRuO_3_ ultrathin films possess in‐plane easy axes, and reveal that the observed in‐plane AHE is an off‐diagonal response intrinsically coupled to the in‐plane spin magnetization through higher‐order terms allowed by trigonal distortion of the films. Appearance of a non‐zero in‐plane anomalous Hall conductivity along with an out‐of‐plane orbital magnetization has been also clarified through symmetry analysis and first‐principles calculation.

## 2 Results

### 2.1 Out‐of‐Plane and In‐Plane AHE

**Figure 2:**
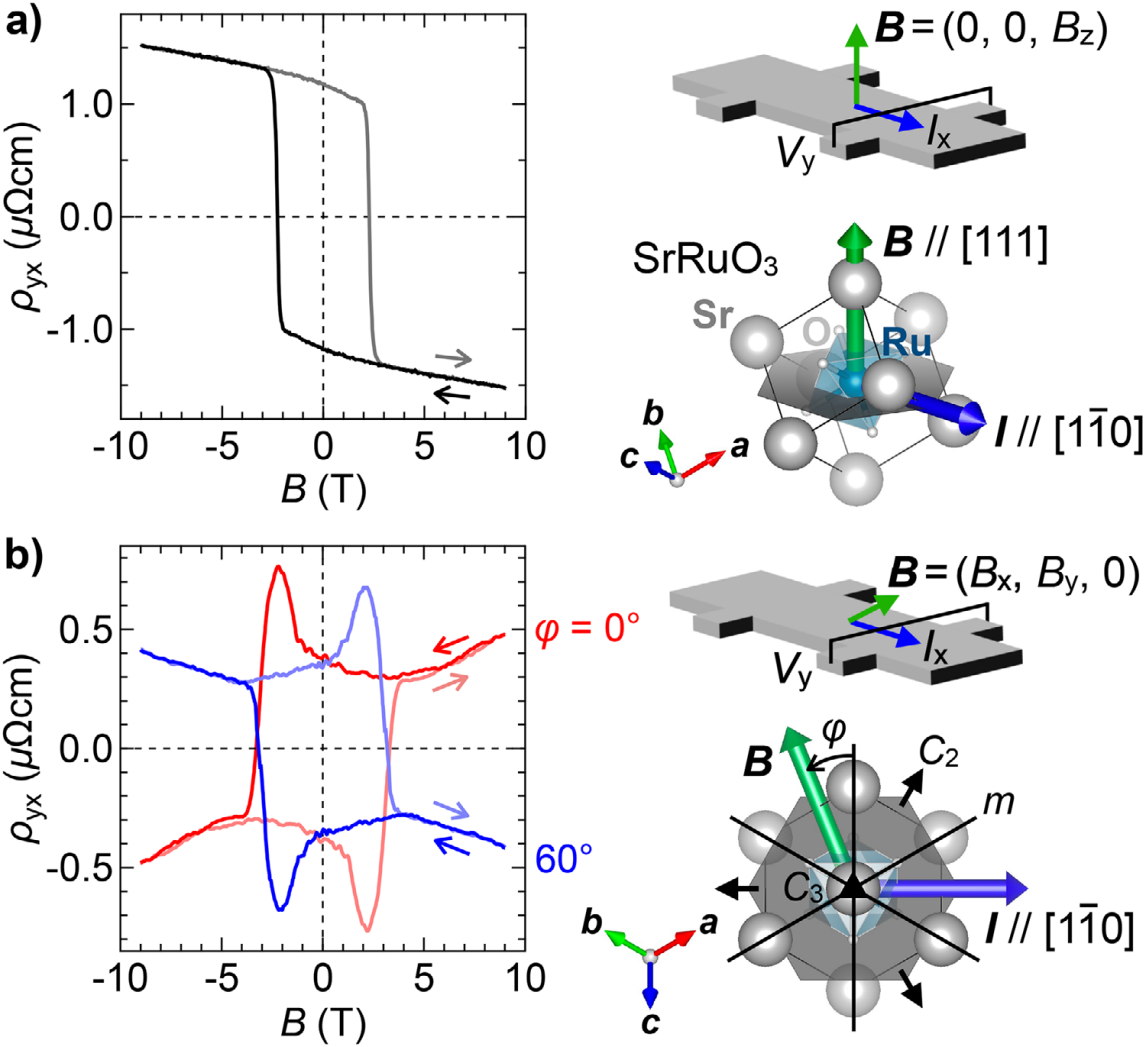
Out‐of‐plane and in‐plane AHE with a hysteresis loop. a) Hall resistivity ρ_yx_ of a (111) SrRuO_3_ film with thickness of 4.1 nm (sample A), taken with sweeping the out‐of‐plane magnetic field along the [111] direction at 2 K. Schematic illustration of the measurement configuration and its correspondence to the SrRuO_3_ crystal structure are shown in the right panel. b) ρ_yx_ measured with sweeping the in‐plane field at azimuthal angles φ = 0° and 60°, where φ is measured from the $\left[11\bar{2}\right]$ direction. Illustration of the in‐plane measurement configuration and its correspondence to the crystal structure are similarly shown, together with fundamental symmetry elements of the *C*_2_ and *C*_3_ rotation axes and the mirror planes.

**Figure** **2**a shows conventional Hall resistivity ρ_yx_ measured for a (111) SrRuO_3_ film with thickness of 4.1 nm (sample A) with sweeping the out‐of‐plane magnetic field at 2 K. The Curie temperature *T*_C_ of this sample is determined to be 130 K, which is slightly lower than the bulk value but consistent with previous studies on (111) SrRuO_3_ thin films (see Section S2 and Figure S2, Supporting Information, for other fundamental transport).^[^ ^25^, ^26^, ^27^ ^]^ ρ_yx_ shows a large hysteresis loop characteristic to hard ferromagnets, also in good agreement with the previous studies. ^[^ ^25^, ^26^, ^27^ ^]^ The slope above the coercive field is roughly 0.03 µΩcmT^−1^.

AHE has not been fully examined in ferromagnets with in‐plane spin magnetization. As shown in Figure 2b, however, ρ_yx_ taken with sweeping the in‐plane [$11\bar{2}$] field at φ = 0° exhibits significantly large values comparable to the out‐of‐plane scan, and continues to increase above the coercive field with a similar slope of 0.03 µΩcmT^−1^. Moreover, ρ_yx_ in the in‐plane scan also remains finite at zero magnetic field accompanied with large hysteresis. Importantly, the present SrRuO_3_ ultrathin films possess in‐plane easy axes as shown later with the field angle dependence, and the observed AHE cannot be explained by out‐of‐plane canting of spin magnetization. The observation rather highlights the importance of out‐of‐plane orbital magnetization coupled to the in‐plane spin magnetization.

ρ_yx_ taken at φ = 60° shows similar in‐plane field dependence with signs opposite to the φ = 0° case. These observations are also consistent with symmetry of the trigonally distorted (111) SrRuO_3_ thin films, which have a *C*_3_ axis along the [111] direction, *C*_2_ axes along the $\left[1\bar{1}0\right]$ and its equivalent directions, and mirror planes on the $\left(1\bar{1}0\right)$ and its equivalent planes. This allows the emergence of finite in‐plane AHE with opposite signs centered at φ = 0°, 120°, 240° and φ = 60°, 180°, 300°.^[^ ^16^, ^18^ ^]^

### 2.2 Azimuthal Angle Dependence

**Figure 3:**
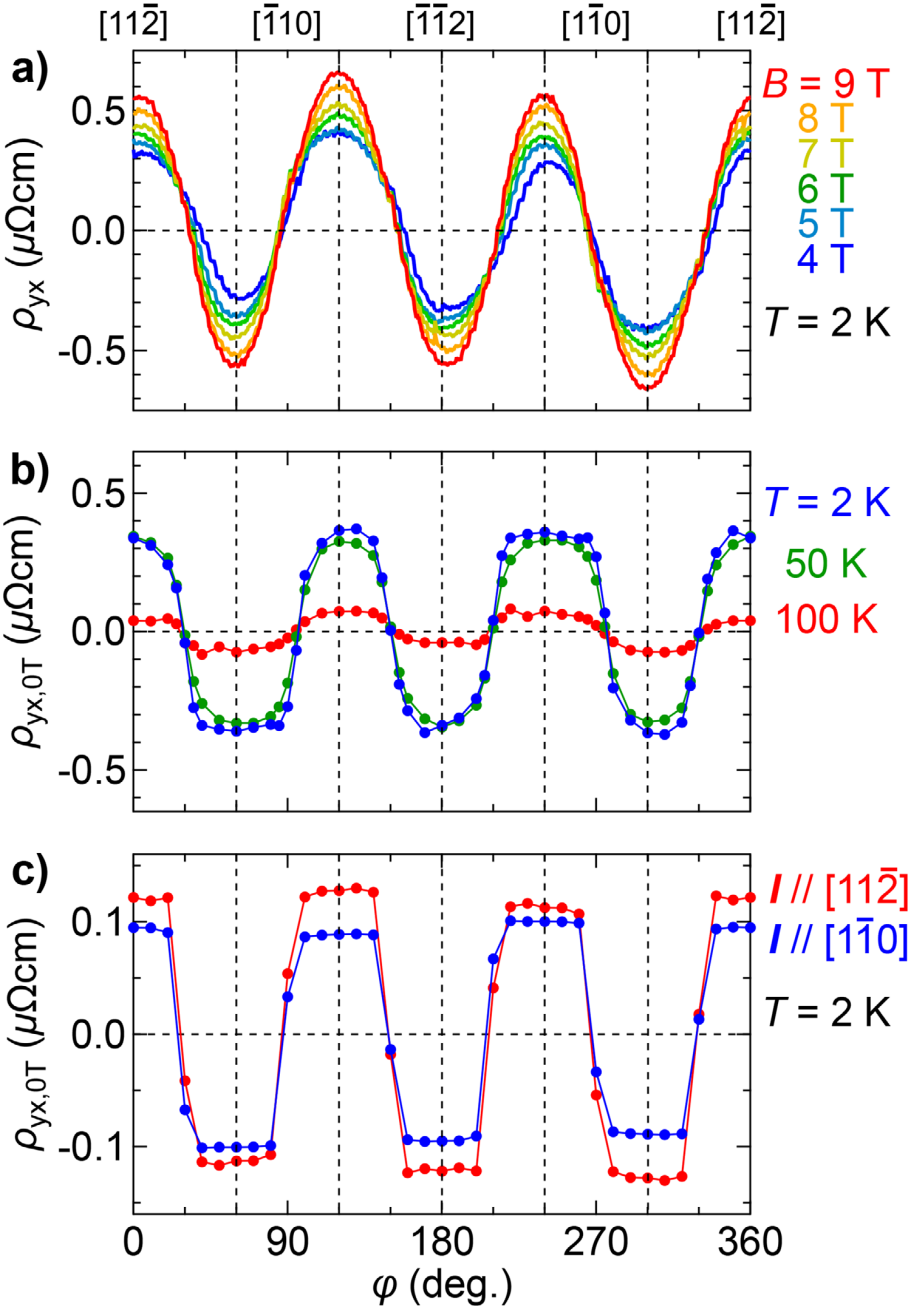
In‐plane AHE at zero magnetic field. a) ρ_yx_ of the SrRuO_3_ film (sample A), taken with continuously changing φ of various in‐plane magnetic fields at 2 K. b) Hall resistivity at zero magnetic field ρ_yx, 0T_, measured after applying the in‐plane field of 9 T and then lowering it to 0 T at each φ. The measurements were performed at 2, 50, and 100 K. c) ρ_yx, 0T_ measured for another (111) SrRuO_3_ film with thickness of 7.0 nm (sample B) after the same magnetization process at 2 K. The measurements were performed on two Hall bar devices where the electric current flows along the $\left[1\bar{1}0\right]$ and $\left[11\bar{2}\right]$ directions, respectively.

**Figure** **3**a demonstrates φ dependence of ρ_yx_, taken with rotating the in‐plane magnetic field on the (111) plane for sample A. ρ_yx_(φ) is derived by antisymmetrization of the raw data ρ_yx, raw_, as expressed by ρ_yx_(φ) = (ρ_yx, raw_(φ) − ρ_yx, raw_(φ + 180°))/2. When the field is above the in‐plane coercive field of about 3 T, ρ_yx_(φ) exhibits a sinusoidal curve with three‐fold symmetry, which is similar to the in‐plane AHE reported for antiferromagnets and soft ferromagnets.^[^ ^5^, ^6^, ^7^ ^]^ Its magnitudes and signs at φ = 0° and 60° are also consistent with the in‐plane field scan data. We note that the slight misalignment of the field with respect to the in‐plane rotation plane results in an out‐of‐plane field component and out‐of‐plane Hall effects which appear with one‐fold symmetry. In the present case, due to the large response of in‐plane AHE, its threefold symmetric pattern is dominantly seen in ρ_yx_ even without subtracting such misalignment‐induced one‐fold component.

To investigate φ dependence of the Hall resistivity at zero field ρ_yx, 0T_, we repeatedly performed a procedure at each φ, which involves increasing the in‐plane field to 9 T, returning it to 0 T, and then measuring the Hall resistivity. Here ρ_yx, 0T_(φ) is obtained by antisymmetrization of a pair of raw data ρ_yx, 0T, raw_(φ) and ρ_yx, 0T, raw_(φ + 180°). As shown in Figure 3b, ρ_yx, 0T_ exhibits a rather square‐wave curve with threefold symmetry for rotation of the polarizing field, taking values consistent with the field scans at φ = 0° and 60° in Figure 2b. ρ_yx, 0T_ decreases with increase in temperature and disappears at *T*_C_, confirming that this response is indeed related to the ferromagnetic ordering in SrRuO_3_ (see Figure S3, Supporting Information, for its detailed temperature dependence). Similar square‐wave curves are reproducibly confirmed in ρ_yx, 0T_ taken for another (111) SrRuO_3_ film with thickness of 7.0 nm (sample B) shown in Figure 3c, regardless of the current direction on the plane. Difference of the φ dependence between the sinusoidal wave at 9 T and the square‐like wave at 0 T suggests the presence of magnetic anisotropy with in‐plane easy axes pointing to the $\left[11\bar{2}\right]$ and its equivalent directions.

### 2.3 Polar Angle Dependence

**Figure 4:**
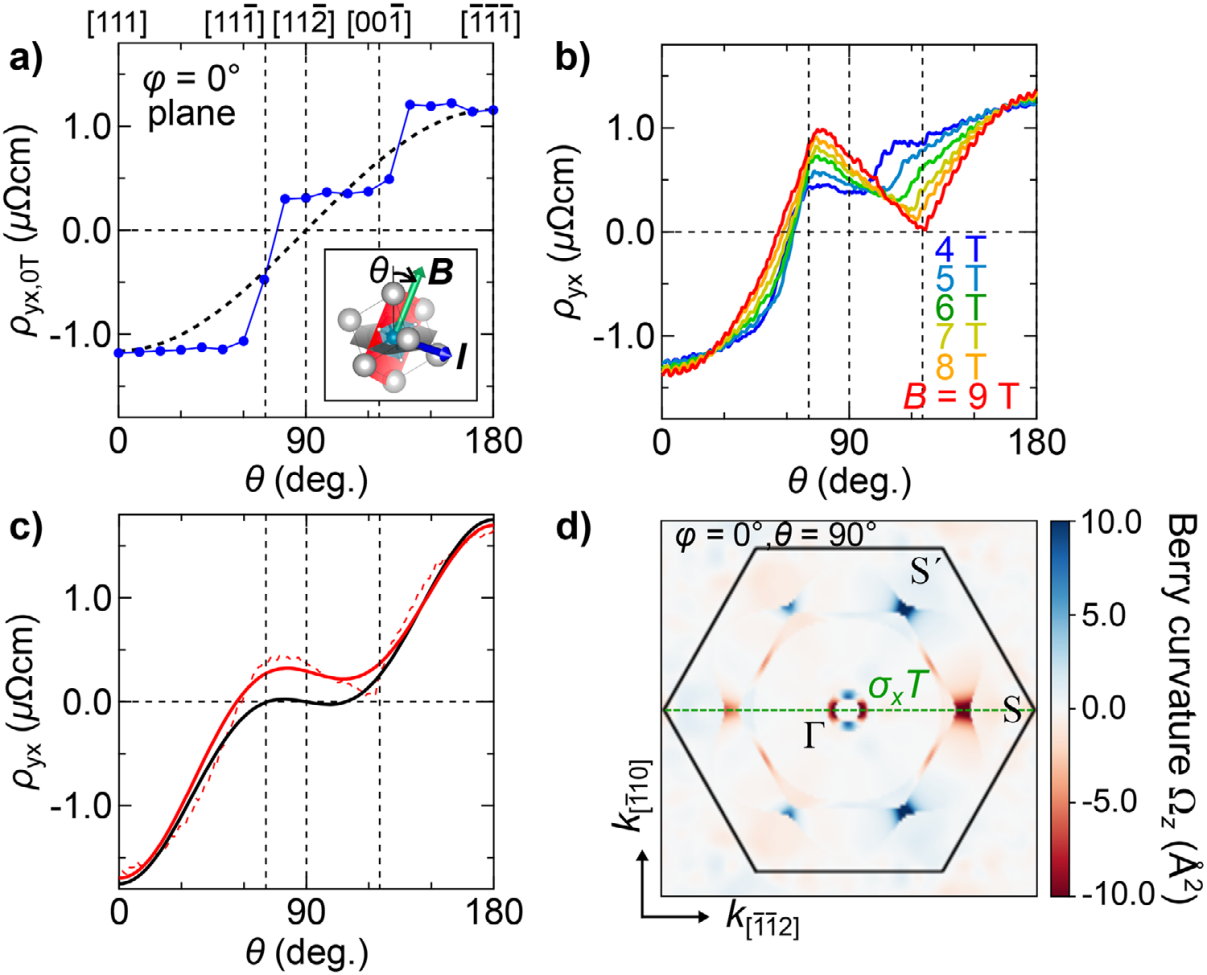
Nonmonotonic polar angle dependence of AHE. a) ρ_yx, 0T_ of the SrRuO_3_ film (sample A), measured after applying the field of 9 T and then lowering it to 0 T at each polar angle θ on the φ = 0° plane at 2 K. θ is measured from the out‐of‐plane [111] direction. b) Comparison of ρ_yx_(θ) measured at various magnetic fields. Only the forward sweeps from $\left[\bar{1}\bar{1}\bar{1}\right]$ to [111] are shown. c) Fitting of ρ_yx_(θ) measured at 9 T (dashed curve) with the theoretical θ dependence obtained from Equation (1). The red solid curve shows the fitting result with the in‐plane AHE term, which yields σ_0_ + σ_2_ = 0.22 × 10^6^ S cm^−1^, σ_1_ = −0.21 × 10^6^ S cm^−1^, and σ_3_ = −1.45 × 10^6^ S cm^−1^ by setting *M* (or *B*) = 1. The black solid curves shows the fitting result without the in‐plane AHE term, which fails to explain the non‐zero anomalous Hall conductivity at θ = 90°. d) Distribution of out‐of‐plane Berry curvature component Ω_*z*_ on the (111) plane calculated with the spin magnetization set to the in‐plane $\left[11\bar{2}\right]$ (φ = 0°, θ = 90°) direction. The magnetic point group with only antiunitary symmetry such as σ_*x*_ *T* perpendicular to $\left[1\bar{1}0\right]$ (dashed line) allows uncancelled Ω_*z*_ when integrated over the Brillouin zone.

To further clarify the magnetic anisotropy near zero field, we present θ dependence of ρ_yx, 0T_ measured on the $\left(\bar{1}10\right)$ or φ = 0° plane in **Figure** **4**a. Here ρ_yx, 0T_(θ) is derived by antisymmetrization of ρ_yx, 0T, raw_(θ) and ρ_yx, 0T, raw_(θ + 180°). ρ_yx, 0T_(θ) exhibits a plateau structure not only around the out‐of‐plane [111] and $\left[\bar{1}\bar{1}\bar{1}\right]$ directions but also over the range including the in‐plane $\left[11\bar{2}\right]$ direction. In addition, the θ scans taken at various magnetic fields (see Figure S4, Supporting Information) reveal that ρ_yx_(θ) exhibits a pronounced hysteresis loop around the $\left[00\bar{1}\right]$ and $\left[11\bar{1}\right]$ directions while the hysteresis loop is closed around the in‐plane $\left[11\bar{2}\right]$ direction. All of these observations evidence that the SrRuO_3_ ultrathin film possesses relatively strong in‐plane shape magnetic anisotropy in addition to 〈111〉 magnetocrystalline anisotropy, realizing a ferromagnetic state with the spins aligned to the in‐plane $\left[11\bar{2}\right]$ direction when the θ is close to 90°. Therefore, the observed spontaneous in‐plane AHE is not due to out‐of‐plane canting of the spin magnetization, and it can be regarded as out‐of‐plane orbital ferromagnetism off‐diagonally coupled to the in‐plane spin magnetization.

Figure 4b compares θ scans measured at various magnetic fields for a forward sweep from $\left[\bar{1}\bar{1}\bar{1}\right]$ to [111] on the φ = 0° plane (see also Figures S4 and S5, Supporting Information, for both the sweeps at various fields and θ scans performed on the φ = 60° plane). While the effect of in‐plane magnetic anisotropy is pronounced at the low fields, ρ_yx_(θ) measured at high fields such as above 6 T exhibits negligibly small hysteresis, indicating that the spin magnetization almost follows the applied field direction during the sweep. ρ_yx_(θ) at high fields exhibits nonmonotonic dependence accompanied by local minimum and maximum around $\left[11\bar{1}\right]$ and $\left[00\bar{1}\right]$ directions, respectively. In particular, the local maximum around $\left[11\bar{1}\right]$ exhibiting a positive sign, which is opposite to the sign at [111]. This sign change feature strongly indicates the necessity of considering a higher‐order effect due to magnetic field *B* ^[^ ^6^ ^]^ or magnetization *M*,^[^ ^7^ ^]^ which includes the in‐plane AHE induced by the in‐plane component of *B* or *M*.

## 3 Discussion

By the structural characterization presented in Section S1 and Figure S1 in Supporting Information, it is clarified that the (111) SrRuO_3_ ultrathin film grown on (111) SrTiO_3_ substrate is subject to trigonal distortion. This distortion modifies the point group of SrRuO_3_ to *D*_3*d*_. Considering all the terms symmetrically allowed by the *D*_3*d*_ point group as well as the translational symmetry, generic formula of anomalous Hall conductivity σ_xy_ with the presence of arbitrary spin magnetization direction (*M*_*x*_, *M*_*y*_, *M*_*z*_) can be formulated as follows,

(1) $$\sigma_{xy}=\sigma_{0}M_{z}+\sigma_{1}\left(3M_{x}^{2}M_{y}-M_{y}^{3}\right)+\sigma_{2}M^{2}M_{z}+\sigma_{3}M_{z}^{3}$$

Here, the *xyz* coordinates is taken with the (111) plane as the reference; namely $x||[1\bar{1}0]$, $y||[11\bar{2}]$, and *z*||[111]. We note that Equation (1) also reflects the magnetic point group when specific *M*_*x*_, *M*_*y*_, *M*_*z*_ components are given. The first term in Equation (1) is the conventional out‐of‐plane AHE, which is induced by the out‐of‐plane spin magnetization component (*M*_*z*_). The second term corresponds to the in‐plane AHE induced by the in‐plane spin magnetization component (*M*_*x*_, *M*_*y*_). The third term corresponds to a correction first pointed out by Kondo,^[^ ^28^ ^]^ and the fourth term is a generalization of the Kondo term that generally appears from the spin‐orbit interaction of itinerant electrons.^[^ ^29^, ^30^ ^]^ From Equation (1), it is clear that when the spin magnetization lies within the plane (*M*_*z*_ = 0), the only term which can contribute to the Hall conductivity is the in‐plane AHE term.

In Figure 4c, we compare the experimental θ‐scan data measured at 9 T with the theoretical θ dependences. The solid curve in red shows the case with the in‐plane AHE term and the one in black shows the case without it. The experimental θ‐scan data is shown by the dashed curve. We note that the spin magnetization *M* almost follows the magnetic field *B* under 9 T, and *M* = (*M*_*x*_, *M*_*y*_, *M*_*z*_) in Equation (1) is effectively replaced with *B* = (*B*_*x*_, *B*_*y*_, *B*_*z*_). The overall shape including the non‐monotonic behavior is well reproduced by Equation (1). It is also clear that the non‐zero Hall conductivity remaining at θ = 90° can be explained only when the in‐plane AHE term is included. The present SrRuO_3_ ultrathin film realizes an in‐plane ferromagnetic state even after switching off the in‐plane field applied around θ = 90°. Therefore, we can also conclude that the spontaneous Hall conductivity at zero field can be similarly understood based on the in‐plane AHE term.

In order to theoretically quantify the in‐plane anomalous Hall conductivity in the present trigonally‐distorted SrRuO_3_, we have also conducted the first‐principles calculation (See Experimental Section for the computational details). When the spin magnetization is oriented along the in‐plane $\left[11\bar{2}\right]$ direction (φ = 0°, θ = 90°), the band structure of SrRuO_3_ hosts type‐II Weyl point pairs formed between the bands dispersing with large bandwidths and a narrow energy gap, which make a significant contribution to the Berry curvature in addition to those from the other trivial bands (See Supporting Information). Figure 4d presents the color map of out‐of‐plane Berry curvature component Ω_*z*_ on the (111) plane. The magnetic point group 2′/*m*′ resulting from in‐plane $\left[11\bar{2}\right]$ spin alignment contains no unitary *C*_2_ rotations along in‐plane directions or vertical mirror operations. Instead, only antiunitary operations such as *C*_2_ *T* or σ_*x*_ *T* are preserved, which allows uncancelled Ω_*z*_ distribution over the Fermi surface and hence non‐zero anomalous Hall conductivity. This is behind the appearance of AHE even with the in‐plane spin magnetization, which is also captured by the phenomenological formula presented in Equation (1), where *M*_*y*_ ≠ 0 gives non‐zero σ_xy_. The in‐plane anomalous Hall conductivity is calculated to be σ_xy_ = 5 S cm^−1^, the sign of which is consistent sign with that of the experimental conductivity σ_xy_ = 0.6 S cm^−1^. We note that the discrepancy in magnitude is probably due to rather low longitudinal conductivity of the present ultrathin films, which are located in the dirty metal regime with the σ_xy_ ∝ σ_xx_^1.6^ scaling.^[^ ^31^ ^]^

One remaining question is the non‐monotonic peak feature around the coercive field observed only for the field scan of in‐plane AHE but not for that of out‐of‐plane AHE as presented in Figure 2. As suggested in previous studies on the thinner films of SrRuO_3_,^[^ ^32^, ^33^ ^]^ inhomogeneity can lead to superposition of out‐of‐plane AHE with different coercive fields, resulting in a non‐monotonic peak feature. While one possibility in the present case is that the in‐plane magnetization and Hall response is more sensitive to possible inhomogeneity in the SrRuO_3_ ultrathin film than the out‐of‐plane counterparts, it requires further studies for clarifying the detailed mechanism.

## 4 Conclusion 

In summary, we demonstrate spontaneous in‐plane AHE in (111)‐oriented ultrathin films of a hard ferromagnet SrRuO_3_. Reflecting the crystal symmetry, the in‐plane AHE emerges with three‐fold symmetry for the field rotation on the Hall deflection plane. Off‐diagonally coupled to the in‐plane spin magnetization, the spontaneous anomalous Hall conduction and associated out‐of‐plane orbital magnetization persist even at zero field, and their sign can be switched via coupling to the in‐plane spin magnetization. Moreover, the polar angle scans reveal the peculiar nonmonotonic behavior which suggests the contribution of the higher‐order terms allowed under the trigonal distortion. The spontaneous emergence of anomalous Hall conductivity and out‐of‐plane orbital magnetization induced by in‐plane spin magnetization has been also theoretically supported by the symmetry consideration based on the magnetic point group and by the first‐principles calculation. Our observations highlight the unique and spontaneous appearance of in‐plane AHE reflecting the crystal symmetry, magnetic hysteresis, and magnetic anisotropy in the conventional ferromagnet. The present work broadens the horizons of Hall physics by demonstrating essential control of the Hall conduction by the in‐plane magnetic field and its history. The off‐diagonal coupling between the orbital and spin magnetization in spontaneous in‐plane AHE may also provide unique functionalities in spintronics and Hall sensor applications.

## 5 Experimental Section

### Epitaxial Film Growth

(111)‐oriented SrRuO_3_ films were grown on (111) SrTiO_3_ substrates in an Eiko EB‐9000S oxide molecular beam epitaxy chamber equipped with a semiconductor‐laser heating system.^[^ ^34^, ^35^ ^]^ SrTiO_3_ substrates were annealed at 870 °C prior to the growth, and then SrRuO_3_ films were grown at 650 °C by supplying 4N Sr from a conventional Knudsen cell, 3N5 Ru from an electron beam evaporator, and O_3_ (60%) + O_2_ (40%) from a Meidensya MPOG‐RDE01C ozone generator. The film thickness was typically designed at ≈4 nm.

### Magnetotransport Measurements

Longitudinal resistivity ρ_xx_ and Hall resistivity ρ_yx_ on Hall bar devices were measured using the conventional low‐frequency lock‐in technique. Field angle dependencies of ρ_xx_ and ρ_yx_ up to 9 T were measured using a sample rotator in a Cryomagnetics cryostat system equipped with a superconducting magnet. The magnetotransport measurements were performed with changing the magnetic field direction within the (111) plane with an azimuthal angle φ (measured from [11$\bar{2}$]), and also from the out‐of‐plane [111] direction toward an in‐plane one with a polar angle θ (measured from [111]).

### First‐Principles Caluclation

First‐principles calculations of the SrRuO_3_ were performed based on the density functional theory (DFT) + U with the generalized gradient approximation in the form of Perdew–Burke–Ernzerhof.^[^ ^36^, ^37^ ^]^ The highly accurate projector‐augmented wave (PAW) method,^[^ ^38^ ^]^ as implemented in the Vienna ab initio simulation package (VASP),^[^ ^39^, ^40^ ^]^ was used. A large plane‐wave cutoff energy of 550 eV was used, and a Γ‐centered 13 × 13 × 13 k‐mesh was adopted for the Brillouin zone integration. The rotationally invariant approach to the DFT+U^[^ ^41^ ^]^ was applied with U = 2.5 eV and J = 0.5 eV on the Ru *d* orbitals. The experimental lattice parameters and atomic positions for the trigonally distorted bulk SrRuO_3_ were used with values of *a* = *b* = *c* = 3.91988 Å and α = β = γ = 89.5658°. The band structures were plotted with the help of VASPKIT,^[^ ^42^ ^]^ and the crystal structures were visualized with VESTA.^[^ ^43^ ^]^ Based on the DFT+U electronic band structures, the Wannier functions were constructed using 40 orbitals including Ru *p*, *d*, and O *s*, *p* orbitals.^[^ ^44^ ^]^ Finally, the intrinsic contribution of the anomalous Hall conductivity was evaluated by the Brillouin zone integration of Berry curvature under the Wannier interpolation.^[^ ^44^, ^45^ ^]^ A fine mesh of 250 × 250 × 250 k points was applied during the integration with good convergence. Furthermore, symmetrization was applied to the Hamiltonian with WannSymm^[^ ^46^ ^]^ according to the magnetic point group for the accurate k‐resolved Berry curvature and orbital magnetization plotted with WannierTools.^[^ ^47^ ^]^

## Conflict of Interest

The authors declare no conflict of interest.

## Author Contributions

M.U. conceived the project and designed the experiments. Y.Ma. and H.K. grew films and performed transport measurements with S.N. and Y.Mu. Y.Ma., H.K., H.S., and S.N. analyzed the data. M.J. performed the first‐principles calculation. R.A. and H.I. jointly discussed the results. M.U. and S.N. wrote the manuscript with input from all authors. All authors have approved the final version of the manuscript.

## Competing Interests

The authors declare that they have no competing interests.

## Data and Materials Availability

The data that support the findings of this study are available from the corresponding author upon reasonable request.

## Supporting information

Supporting Information
