# Supplementary material for: Spontaneous In‐Plane Anomalous Hall Response Observed in a Ferromagnetic Oxide
Source: Adv Mater. 2025 Sep 16;37(47):e02624. doi: 10.1002/adma.202502624 (PMC12651109; doi:10.1002/adma.202502624)
Supplement: Supplementary file 1 — Supporting Information [file ADMA-37-e02624-s001.pdf]

ADVANCED MATERIALS

Supporting Information

for *Adv. Mater.*, DOI 10.1002/adma.202502624

Spontaneous In-Plane Anomalous Hall Response Observed in a Ferromagnetic Oxide

*Shinichi Nishihaya, Yuta Matsuki, Haruto Kaminakamura, Hiroki Sugeno, Ming-Chun Jiang, Yoshiya Murakami, Ryotaro Arita, Hiroaki Ishizuka and Masaki Uchida**

Supporting Information for
Spontaneous In-Plane Anomalous Hall Response Observed in a Ferromagnetic Oxide

Shinichi Nishihaya[1], Yuta Matsuki[1], Haruto Kaminakamura[1], Hiroki Sugeno[1], Ming-Chun Jiang[2,3], Yoshiya Murakami[1], Ryotaro Arita[2,4], Hiroaki Ishizuka[1], Masaki Uchida[1,5,*]

[1]Department of Physics, Institute of Science Tokyo, Tokyo 152-8551, Japan

[2]RIKEN Center for Emergent Matter Science, 2-1 Hirosawa, Wako 351-0198, Japan

[3]Department of Physics and Center for Theoretical Physics, National Taiwan University, Taipei 10617, Taiwan

[4]Department of Physics, University of Tokyo, 7-3-1 Hongo, Bunkyo-ku, Tokyo 113-0033, Japan

[5]Toyota Physical and Chemical Research Institute, Nagakute 480-1192, Japan

[*]To whom correspondence should be addressed: E-mail: m.uchida@phys.sci.isct.ac.jp

S1 Structural characterization of a (111) $SrRuO_3$ film

Figures S1a and S1b summarize the reciprocal space mappings around (332) and (312) Bragg peaks measured on a 58 nm thick (111) $SrRuO_3$ film. Figures S1c presents the φ-scans taken around the (332) Bragg peak. Even for the film as thick as 58 nm, the in-plane lattice constant of $SrRuO_3$ along both $[11\bar{2}]$ and $[1\bar{1}0]$ directions matches that of $SrTiO_3$ substrate, indicating that the SRO film is fully strained on the (111) $SrTiO_3$ plane. $SrRuO_3$ is subject to the trigonal distortion with 0.53% shrinkage along $[11\bar{2}]$ and 0.60% elongation along [111] as illustrated in Figure S1d. It is reasonable to assume the same coherent strain and trigonal distortion also for the $SrRuO_3$ ultrathin films studied for the in-plane anomalous Hall effect (in-plane AHE) in the main text. It has been reported that such epitaxial strain imposed on the (111) plane is accompanied by significant suppression of the RuO_6 octahedra tilting in orthorombic $SrRuO_3$ bulks[1, 2]. While the (111) $SrRuO_3$ films are trigonally distorted, due to the small magnitude of the epitaxial strain, the pseudocubic expression is adopted for describing the crystal orientation in the present work.

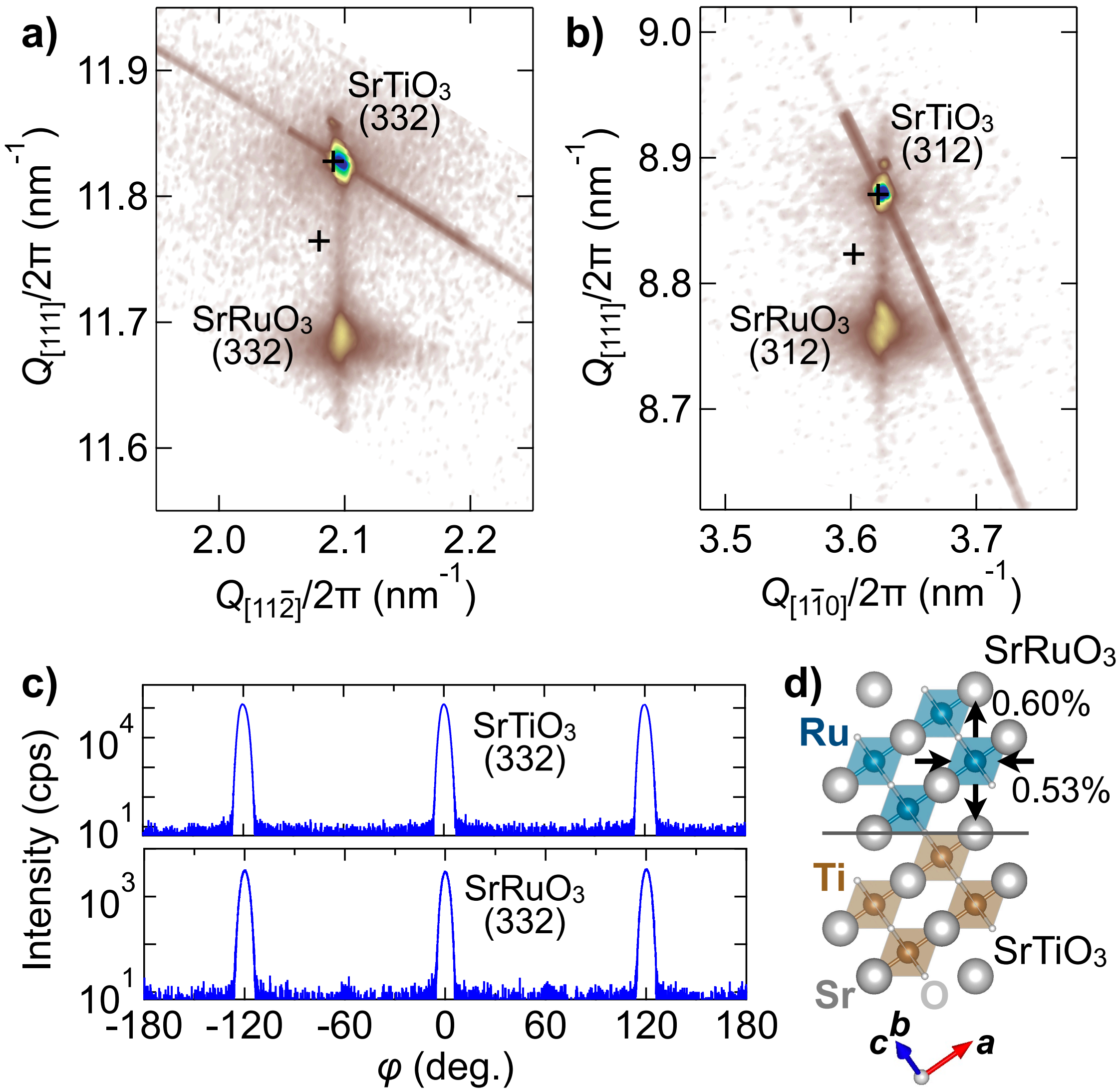

Figure S1. XRD characterization of a (111)-oriented $SrRuO_3$ film. Reciprocal space maps collected around a) (332) and b) (312) Bragg peaks of the $SrTiO_3$ substrate and the $SrRuO_3$ film. The cross mark denotes the Bragg peak position expected from the bulk pseudocubic lattice constant 3.93Å. c) φ-scan taken for the (332) Bragg peak of the $SrTiO_3$ substarte (top) and the $SrRuO_3$ film (bottom), showing a three-fold symmetry. (D) Schematic illustration of the $SrRuO_3/SrTiO_3$ heterostructure viewed from the $[1\bar{1}0]$ direction.

S2 Fundamental transport of a (111) $SrRuO_3$ ultrathin film

Figure S2a presents temperature dependence of the resistivity ρ_{xx} of the 4.1 nm thick $SrRuO_3$ (Sample A). The Curie temperature T_C is determined to be 130 K, which is consistent with previous studies on (111) $SrRuO_3$ thin films [3, 4, 5]. Figure S2b shows the out-of-plane field dependence of ρ_{xx} measured at 2 K. ρ_{xx} exhibits negative magnetoresistance with a butterfly shaped hysteresis loop typically observed in ferromagnetic $SrRuO_3$.

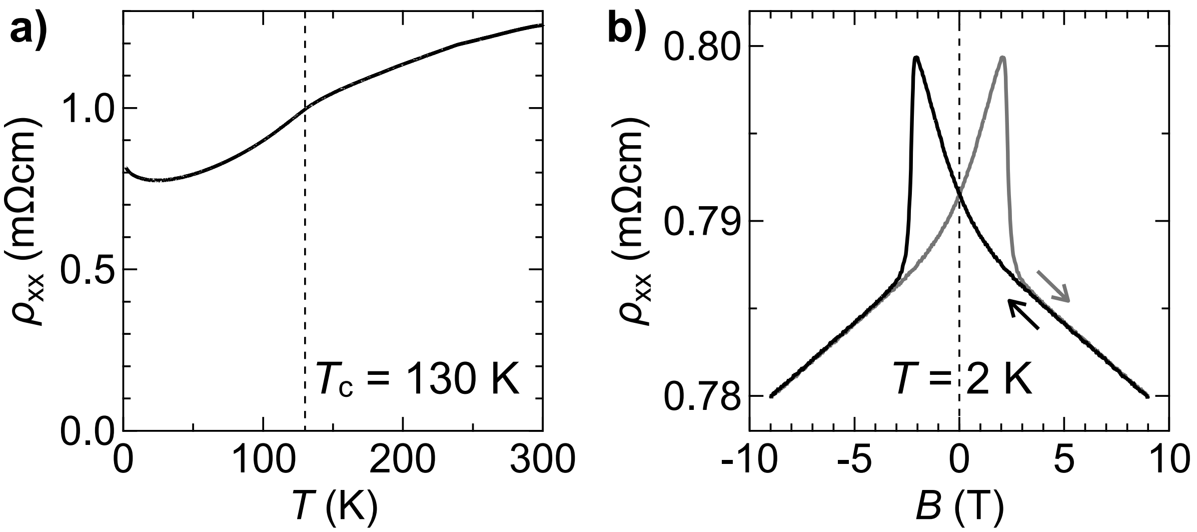

Figure S2. Fundamental transport of a 4.1 nm thick (111) $SrRuO_3$ film. a) Temperature dependence of longitudinal resistivity ρ_{xx}, exhibiting a broad kink at T_C = 130 K. b) Out-of-plane magnetic field dependence of ρ_{xx} at 2 K.

S3 Temperature dependence of Hall response

Figure S3a presents temperature dependence of the zero-field anomalous Hall resistivity $\rho_{\mathrm{yx,0T}}$ measured after increasing the in-plane mangetic field to 9 T and then returning it to 0 T for the in-plane $[11\bar{2}]$ ($\varphi = 0°$) direction at each temperature. $\rho_{\mathrm{yx,0T}}$ is gradually suppressed as increasing temperature toward T_{C}. Figure S3b shows temperature dependence of $\rho_{\mathrm{yx}}(\theta)$ taken for the $\varphi = 0°$ plane at 9 T. With increase in temperature, the nonmonotonic behavior with local minimum and maximum is broadened and suppressed. This is also in line with the temperature dependence of $\rho_{\mathrm{yx,0T}}$ in Figure S3a. These observations confirm the importance of ferromagnetic ordering in the observed in-plane AHE, which induces exchange splitting of bands and formation of several Weyl point pairs in $SrRuO_3$.

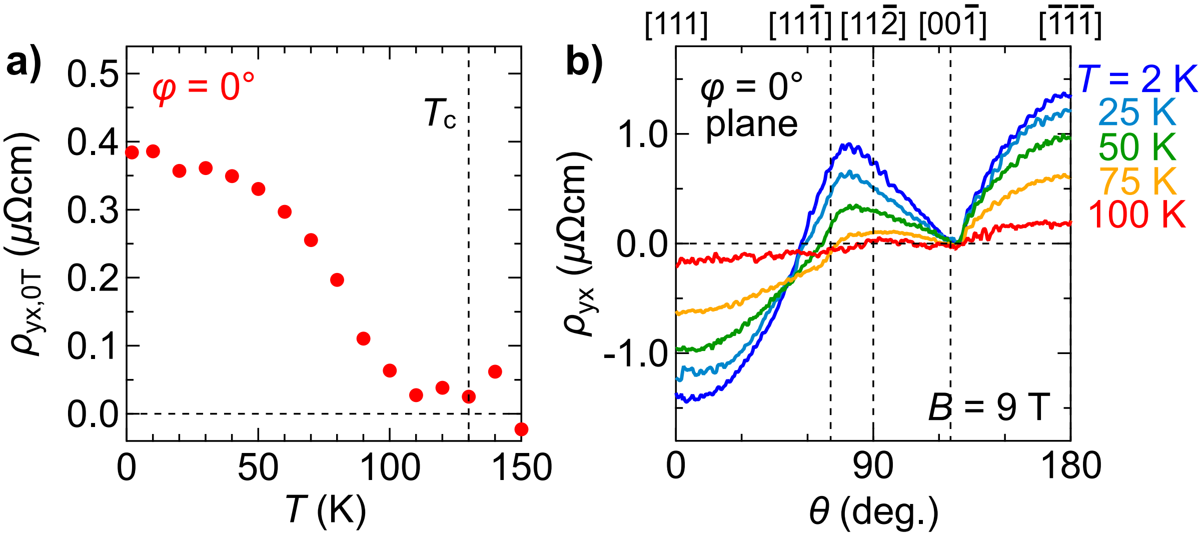

Figure S3. Temperature dependence of Hall response. a) Temperature dependence of zero-field anomalous Hall resistivity $\rho_{\mathrm{yx,0T}}$. $\rho_{\mathrm{yx,0T}}$ is measured after increasing the in-plane mangetic field to 9 T and then returning it to 0 T for the $\varphi = 0°$ direction at each temperature. b) Temperature dependence of the θ scan of ρ_{yx} measured on the $\varphi = 0°$ plane at 9 T.

S4 θ dependence of Hall response

Figure S4 summarizes θ scans of Hall resistivity ρ_{yx} measured with various magnetic fields for the $\varphi = 0°$ plane. Each measurement is performed after applying the field of 9 T to the out-of-plane $[\bar{1}\bar{1}\bar{1}]$ ($\theta = 180°$) direction. At low fields, the effect of magnetic anisotropy becomes evident. For example, $\rho_{\mathrm{yx}}(\theta)$ measured at the fields of 6 T to 4.5 T shown in Figures S4d-S4g, exhibits a pronounced hysteresis loop around $[00\bar{1}]$ and $[11\bar{1}]$ directions, while a plateau structure with vanished hysteresis around the in-plane $[11\bar{2}]$ direction. These observations directly evidence that the $SrRuO_3$ ultrathin film possesses in-plane shape magnetic anisotropy in addition to the $\langle 111 \rangle$ magnetocrystalline anisotropy, and thus the spin magnetization along the easy axis of the $[11\bar{2}]$ direction remains robust down to the zero field when the field is applied near the in-plane direction as shown in Figure 4a. For $\rho_{\mathrm{yx}}(\theta)$ measured at the even lower fields shown in Figures S4h and S4i, the hysteresis loop is further extended between the out-of-plane $[111]$ and $[\bar{1}\bar{1}\bar{1}]$ directions, reflecting the effect of initial application of 9 T field to the out-of-plane $[\bar{1}\bar{1}\bar{1}]$ direction before the θ scans.

On the other hand, $\rho_{\mathrm{yx}}(\theta)$ measured at high fields such as above 6 T shown in Figures S4a-S4c, the spin magnetization almost follows the direction of the applied field, showing only negligibly small hysteresis. The nonmonotonic bahvior of $\rho_{\mathrm{yx}}(\theta)$ observed under these higher fields clarifies that the higher order terms allowed by trigonal distortion of the films is essential for the in-plane AHE as discussed in the main text. Similar nonmonotonic dependence is also confirmed in another θ scan on the $(\bar{1}01)$ or $\varphi = 60°$ plane as shown in Figure S5.

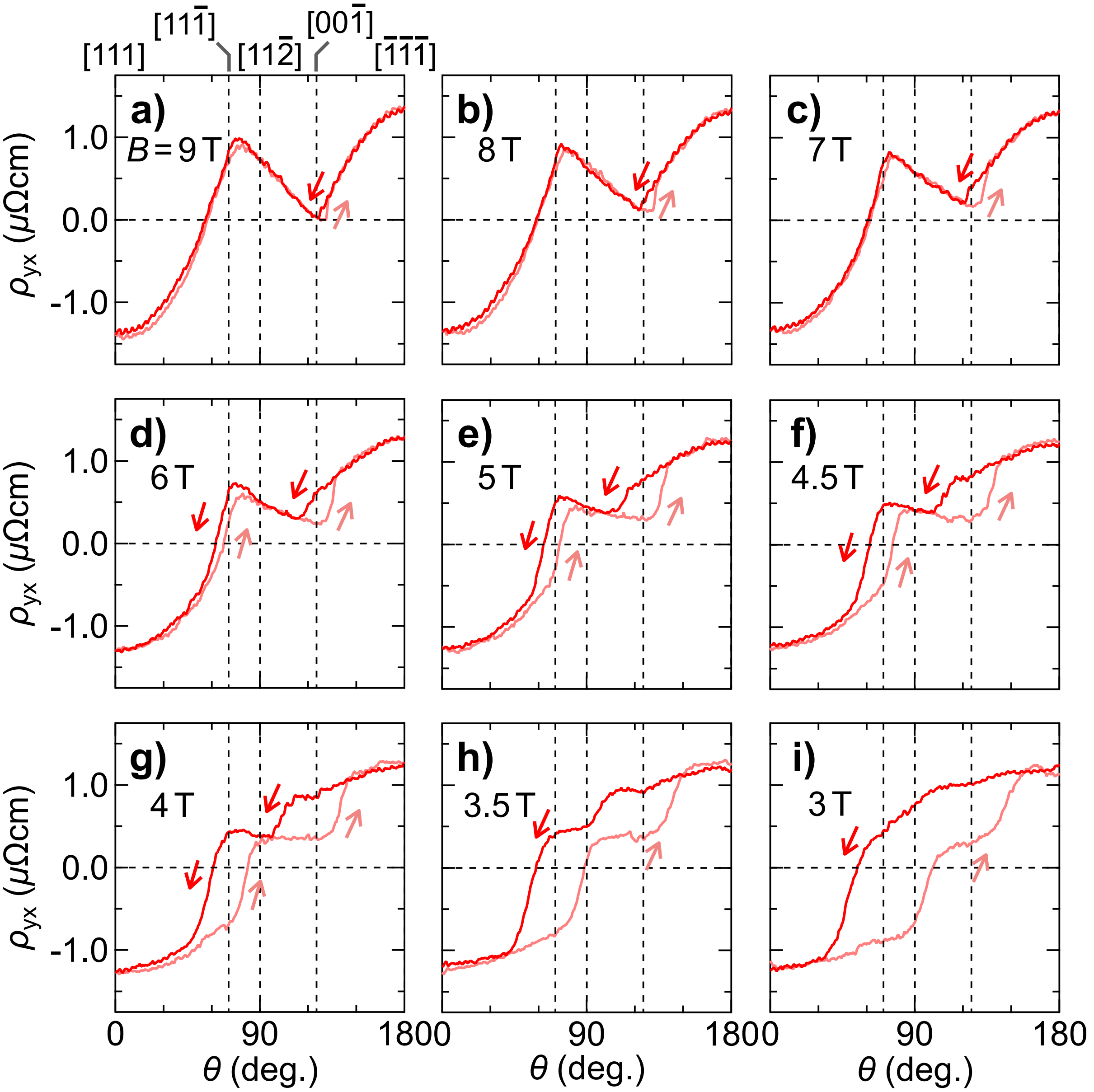

Figure S4. θ scans of Hall resistivity $\rho_{\rm yx}$ measured at various magnetic fields. $\rho_{\rm yx}(\theta)$ curve measured at 2 K and a) 9 T, b) 8 T, c) 7 T, d) 6 T, e) 5 T, f) 4.5 T, g) 4 T, h) 3.5 T, and i) 3 T for the $\varphi = 0°$ plane. Each measurement is performed after applying the field of 9 T to the out-of-plane $[\bar{1}\bar{1}\bar{1}]$ ($\theta = 180°$) direction.

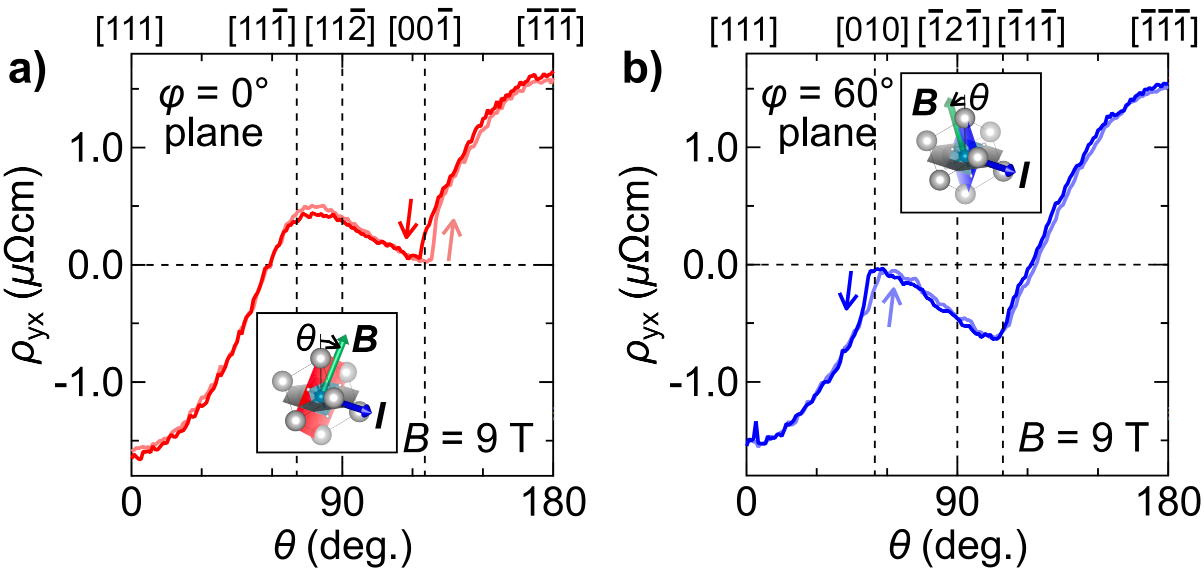

Figure S5. **θ scans of Hall resistivity ρ_{yx} measured on different φ planes.** $\rho_{\mathrm{yx}}(\theta)$ measured on the (A) $\varphi = 0°$ and (B) $60°$ planes at 9 T and 2 K.

S5 First-principles calculation

First-principles calculation was performed to theoretically quantify the in-plane anomalous Hall conductivity in the present trigonally-distorted $SrRuO_3$. The computational details are summarized in Experimental Section in the main text.

Figure S6a presents the band structure and the Berry curvature calculated along the high symmetry lines for the trigonally-distorted $SrRuO_3$ with the spin magnetization M lying in the in-plane $[11\bar{2}]$ direction. Figure S6b depicts the three-dimensional Brillouin zone. The highlighted two bands in orange and pink are representative bands which host Weyl points near the Fermi level. Integration of the Berry curvature component Ω_y and Ω_z corresponds to the Hall conductivity for out-of-plane AHE (σ_{zx}) and in-plane AHE (σ_{xy}) when M points to $[11\bar{2}]$.

Figures S6c and S6d present the band dispersions along the directions where the type-II Weyl point pairs (W1,W2) and (W3,W4) are formed between the two highlighted bands in Figure S6a. The coordinates for the Weyl points are: W1, W2 ($\pm 0.0504, \pm 0.0490, \mp 0.0601$); W3,W4 ($\pm 0.3364, \pm 0.3367, \mp 0.3611$) in reciprocal lattice units. Since the two bands forming the Weyl points are dispersing with large bandwidth and a narrow energy gap between them, the Berry curvature contribution of those Weyl points remains significant even at energies away from the node position. As presented in Figure S6d, particularly large Berry curvature is confirmed around the momentum region where the two parallel bands derived from the Weyl points cross the Fermi level. Since $SrRuO_3$ has a large Fermi surface with multiple Fermi pockets, there are other bands also contributing to the Berry curvature and anomalous Hall conductivity in addition to the Weyl bands as shown in Figures S6a and S6c. Thus, the metallic bands with large bandwidth combined with the tilted dispersion of the type-II Weyl points grant the Berry curvature peaks of $SrRuO_3$.

The Hall conductivities are calculated to be $\sigma_{\mathrm{zx}} = 93$ S/cm and $\sigma_{\mathrm{xy}} = 5$ S/cm for the $M \parallel [11\bar{2}]$ case, and $\sigma_{\mathrm{xy}} = -68$ S/cm for the $M \parallel [111]$ case. The orbital magnetization also shows nonzero component not only for M_y^{orb} but also for M_z^{orb}. On the other hand, the experimentally obtained anomalous Hall conductivities is $\sigma_{\mathrm{xy}} = 0.6$ S/cm for $M \parallel [11\bar{2}]$ (in-

plane AHE) and $\sigma_{\rm xy} = -1.9$ S/cm for $M \parallel [111]$ (out-of-plane AHE). Notably, the sign of $\sigma_{\rm xy}$ between in-plane AHE and out-of-plane AHE is consistent between the theoretical and experimental results. Thus, the first-principles calculation also supports in-plane AHE and out-of-plane orbital magnetization induced by in-plane spin magnetization in the present $SrRuO_3$ films.

Figure S7 presents the distribution of Berry curvature components $\boldsymbol{\Omega} = (\Omega_x, \Omega_y, \Omega_z)$ and orbital magnetization components $\boldsymbol{M}^{\rm orb} = (M_x^{\rm orb}, M_y^{\rm orb}, M_z^{\rm orb})$ on the (111) plane obtained by the first-principles calculation with setting the spin magnetization M to the in-plane $[11\bar{2}]$ direction. The magnetic point group $2'/m'$ realized with $M \parallel [11\bar{2}]$ has the antiunitary symmetry $\sigma_x T$ (denoted by the dashed line in Figures S7a and S7d), which dictates the perfect cancellation of Ω_x and $M_x^{\rm orb}$ when integrated over the Brillouin zone due to $\sigma_x T \Omega_x = -\Omega_x, \sigma_x T M_x^{\rm orb} = -M_x^{\rm orb}$. The other components such as Ω_z and $M_z^{\rm orb}$, on the other hand, remain nonzero even after the integration due to absence of a C_2 rotational symmetry axis along any in-plane directions nor vertical mirror planes.

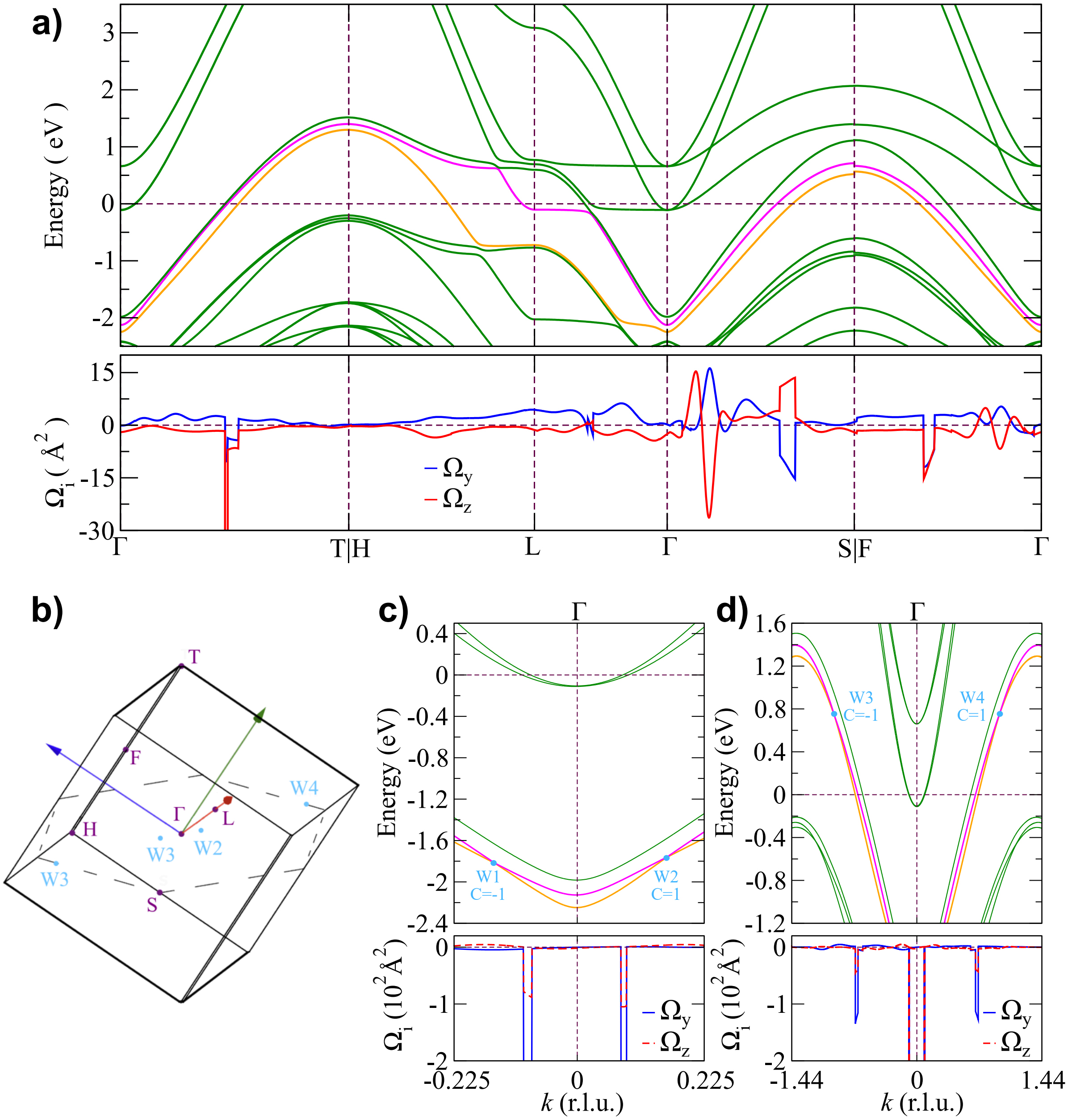

Figure S6. First-principles calculation of band structure of the trigonally distorted $SrRuO_3$. (a) Band structure and Berry curvature distribution calculated along the high symmetry lines. (b) Three-dimensional Brillouin zone illustrated with the representative Weyl point pairs (W1,W2) and (W3,W4) formed between the two bands highlighted in (a) near the Fermi level. The dashed line indicates the two-dimensional slice perpendicular to [111]. Magnified view of the band dispersions and the Berry curvature distribution along the momentum directions with the Weyl point pairs (c) (W1,W2) and (d) (W3,W4).

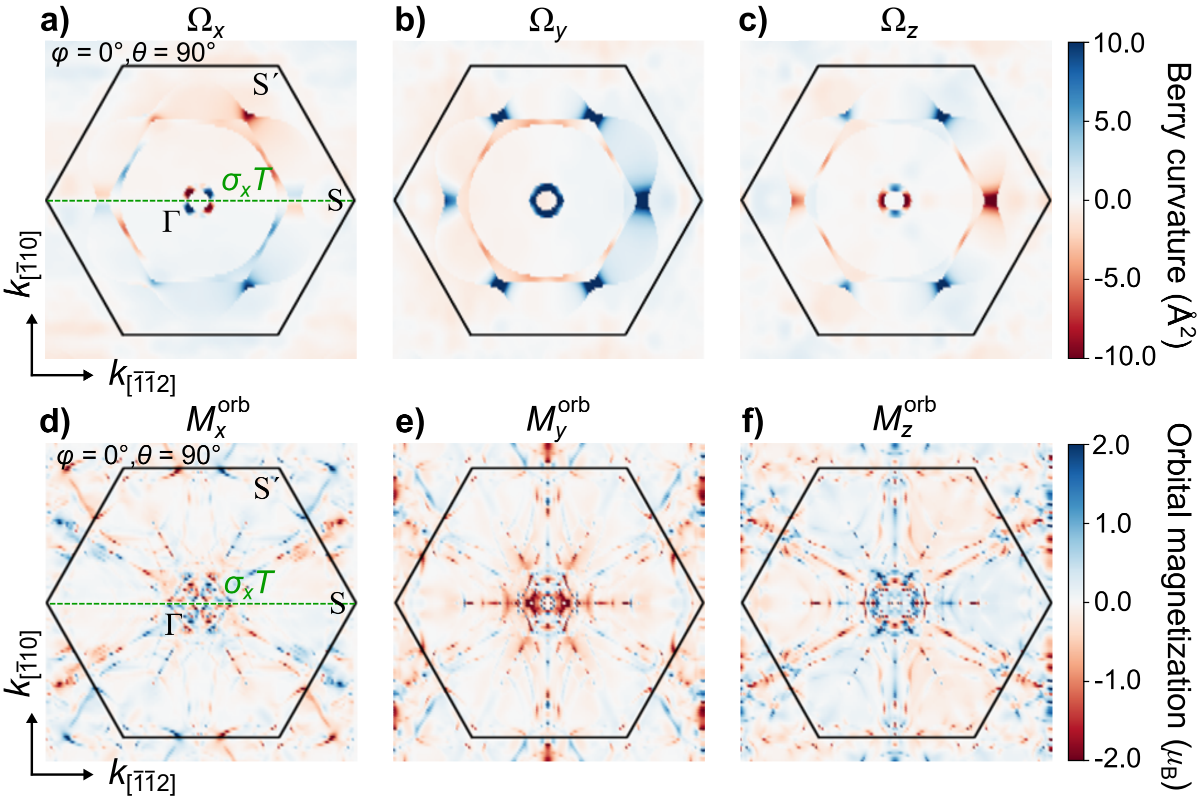

Figure S7. Color maps of Berry curvature and orbital magnetization components on the (111) plane. Distribution of (a)-(c) Berry curvature components $(\Omega_x, \Omega_y, \Omega_z)$ and (d)-(f) orbital magnetization components $(M_x^{\rm orb}, M_y^{\rm orb}, M_z^{\rm orb})$ on the (111) plane calculated with the spin magnetization set to the $y \parallel [11\bar{2}]$ $(\varphi = 0°, \theta = 90°)$ direction.

References

[1] Z. Wang, W. Qi, J. Bi, X. Li, Y. Chen, F. Yang, Y. Cao, L. Gu, Q. Zhang, H. Wang, J. Zhang, J. Guo, X. Liu, *Chin. Phys. B* **2022**, 31, 126801.

[2] Z. Ding, X. Chen, Z. Wang, Q. Zhang, F. Yang, J. Bi, T. Lin, Z. Wang, X. Wu, M. Gu, M. Meng, Y. Cao, L. Gu, J. Zhang, Z. Zhong, X. Liu, J. Guo, *npj Quan. Mater.* **2023**, 8, 43.

[3] W. Lin, L. Liu, Q. Liu, L. Li, X. Shu, C. Li, Q. Xie, P. Jiang, X. Zheng, R. Guo, Z. Lim, S. Zeng, G. Zhou, H. Wang, J. Zhou, P. Yang, Ariando, S. J. Pennycook, X. Xu, Z. Zhong, Z. Wang, J. Chen, *Adv. Mater.* **2021**, 33, 2101316.

[4] H. Ryu, Y. Ishida, B. Kim, J. R. Kim, W. J. Kim, Y. Kohama, S. Imajo, Z. Yang, W. Kyung, S. Hahn, B. Sohn, I. Song, M. Kim, S. Huh, J. Jung, D. Kim, T. W. Noh, S. Das, C. Kim, *Phys. Rev. B* **2020**, 102, 041102(R).

[5] A. Rastogi, M. Brahlek, J. M. Ok, Z. Liao, C. Sohn, S. Feldman, H. N. Lee *APL Mater.* **2019**, 7, 091106.
